# Infection with multiple hepatitis C virus genotypes detected using commercial tests should be confirmed using next generation sequencing

**DOI:** 10.1038/s41598-019-42605-z

**Published:** 2019-06-25

**Authors:** Belén Fernández-Caso, Jose Ángel Fernández-Caballero, Natalia Chueca, Eukene Rojo, Adolfo de Salazar, Luisa García Buey, Laura Cardeñoso, Federico García

**Affiliations:** 1 Servicio de Microbiologia, Hospital Universitario de La Princesa; Instituto de Investigacion Sanitaria La Princesa, Madrid, Spain; 2 Unidad de Gestión Clinica de Microbiologia, Hospital Universitario San Cecilio; Instituto de Investigación Biosanitaria ibs.Granada; Red de Investigación en SIDA, RD16/0025/0040, Granada, Spain; 3 Servicio de Aparato Digestivo - Unidad de Hepatologia, Hospital Universitario de La Princesa; Instituto de Investigacion Sanitaria La Princesa, Madrid, Spain

**Keywords:** Hepatitis C, Hepatitis C virus

## Abstract

Current HCV genotyping methods may have some limitations in detecting mixed infections. We aimed to determine the accuracy of genotyping and the detection of mixed-genotype infections using the Abbott-RealTime HCV Genotype II assay (Abbott-RT-PCR) in comparison with a Roche-Next Generation Sequencing assay (Roche-NGS). Plasma samples collected from 139 HCV-infected patients tested with Abbott-RT-PCR, 114 with single genotype (GT) and 25 with mixed GTs were genotyped using Roche-NGS. Roche-NGS confirmed all single GTs obtained with Abbott-RT-PCR. One case of Abbott GT 4 was found as GT 1a using Roche-NGS. Genotype 5 was confirmed using Roche-NGS in 75% cases (3 out of 4 cases). Twenty-five patients were identified as having mixed HCVinfections using Abbott-RT-PCR. The concordance between Abbott-RT-PCR and Roche-NGS was 76% (19 out of 25 cases). Three mixed-GT infections identified with the Abbott assay (two (1b + 4); one (1a + 3)) were reported as pure 1b using Roche-NGS. Very divergent results were found for the other three samples. When compared to Roche-NGS, Abbott-RT-PCR has performed excellently for the determination of patients infected with single GTs. For patients that are categorized as having a mixed infection using Abbott-RT-PCR, we recommend an NGS assay as a confirmation test.

## Introduction

Hepatitis C virus (HCV) has been classified into seven major genotypes (GTs) (GT 1 – GT 7), with whole-genome nucleotide sequences differing between them by 30% approximately. A total of 67 confirmed subtypes with 15–30%^1^ of nucleotide sequence divergence among them have been described. Recently, an additional HCV GT has been identified in patients from Punjab^2^.

Infection with more than one HCV GT or subtype at the same time, known as mixed-GT infection, has been demonstrated^3–7^. In the general population, mixed HCV GT infection was reported to be present in 2–10% of all HCV positive patients^8^. However, inconsistent data regarding the true prevalence of this phenomenon are observed, likely biased by methodological factors. It has been suggested that mixed HCV GT infections may be much more common in the general population than anticipated. However, screening for infection with multiple HCV GTs is not performed in routine clinical practice and the prevalence of mixed HCV GT infection might be underestimated^9^.

A variety of technologies have been developed for HCV GT and subtype determination^10–18^. So far, several non-sequencing-based HCV genotyping assays, mainly based on reverse hybridization or real-time PCR (RT-PCR), such as the Abbott RealTime HCV Genotype II assay (Abbott RT-PCR), are commercially available and used for routine determination of the HCV GT and selected subtypes. These methods, nevertheless, may have some limitations in detecting mixed infections, as they are designed to identify only the dominant HCV GT in the population and may not detect minor variants^7,19^. For this reason, they may provide unambiguous results failing to assign the HCV GT/subtype.

In recent years, a few sequencing-based HCV genotyping assays have been developed. Currently, Sanger sequencing is still considered a reference method for HCV genotyping, but mixed infections might not be detected by this method, especially if one population shows a low prevalence^20^. In contrast, Next-generation sequencing (NGS) allows to investigate viral heterogeneity at much larger detail: its high throughput generates millions of reads in a single sequencing run, leading to a higher sensitivity compared to the detection of minor strains, which would go undetected by standard sequencing methods^21,22^.

The aim of the present study was to determine the reliability and accuracy on genotyping and the detection of mixed GT infections using a NGS assay of a fragment of the NS5B region (Roche Diagnostics, from now Roche-NGS), and to compare these results with those obtained using the Abbott RT-PCR which is based on real-time PCR amplification of two genomic viral regions (nonstructural protein 5B [NS5B] and the 5′untranslated region [UTR]).

## Materials and Methods

### Patients

Plasma samples obtained from 139 HCV-diagnosed patients initially tested using Abbott RT-PCR were genotyped using Roche-NGS. Samples were collected at the Hospital Universitario de La Princesa. Samples were obtained from HCV-diagnosed patients during a 7-year period, from 2011 to 2017. Sixty-nine males (50%) and seventy females (50%), with a median age of 56 years (range 26–89 years), were recruited. The mean HCV viral load was 6.05 log10 IU/mL (median 6.20; range 2.95–7.52). All specimens were processed as recommended and remaining plasma was stored at −80 °C. The study was conducted in accordance with the ethical principles of the Declaration of Helsinki and it was approved by the Ethics Committee of the Hospital Universitario de La Princesa. Informed consent of patients was not required.

We have analyzed all mixed infections obtained with the routine Abbott RT-PCR during the study period. In addition, to determine the prevalence of mixed HCV GT infections, we performed a case (mixed infection)-control study (1:4) to investigate if Roche-NGS could find additional cases of mixed infection. Therefore, the samples we have tested included the following:

- Single-GT samples: 114 plasma samples from patients infected with HCV GTs 1a, 1b, 1 subtype not determined, 2, 3, 4 or 5.
- Mixed-GT samples: 25 patients containing mixed GTs based on the Abbott RT-PCR assay. “Genotype X reactivity with Y” results were also reported as mixed infections.

### Abbott RT-PCR-based genotyping

The Abbott RT-PCR commercial technique for HCV genotyping used is based on real-time PCR amplification of two genomic viral regions (NS5B and 5′UTR). This technique requires 500 μL of plasma or serum and its limit of detection is 500 IU/mL. The test is carried out in two modules. In the first module, automated sample preparation, RNA extraction and transfer to a 96-well optical reaction plate were carried out. Reagents consist of three master mixes (A, B, and C) dispensed along with aliquots of the nucleic acid samples to the plate. Each processed sample is added to 1 well containing master mix A, 1 well containing master mix B, and 1 well containing master mix C. Then the plate is manually transferred to the second module where RT-PCR takes place. The assay uses four sets of PCR primers. One set of primers targets a sequence within the 5′UTR designed to amplify all HCV isolates. The second primer set is designed to amplify the NS5B region of GT 1a. The third HCV primer set is designed to amplify the NS5B region of GT 1b. The fourth set of primers was used as internal control. The assay detects GTs 1, 2, 3, 4, 5, and 6, and subtypes 1a or 1b, requiring three separate reactions (A reaction for all HCV isolates, subtype 1a and type 3 isolates; B reaction for type 2, subtype 1b and type 1 isolates; C reaction for type 4, type 5 and type 6 isolates). Genotypes are detected using genome specific fluorescent-labeled oligonucleotide probes. Plasma samples were processed in strict accordance with the manufacturer’s instructions.

### Roche NGS-based genotyping

A NGS prototype provided by Roche Diagnostics (Roche-NGS) was used on an Illumina platform with the purpose of analyzing the NS5B region of HCV genomes as previously described^12^.

HCV RNA was extracted using the Magna Pure Compact System (Roche), which utilizes magnetic-bead technology, as recommended by the manufacturer. Then RNA was eluted into 50 μL of kit buffer from sample volumes of 400 or 1000 μL according to availability. Subsequently, RNA was purified using Ampure Agencourt RNAClean XP (Beckman Coulter) before cDNA synthesis.

The method previously described by Quer *et al*.^23^ was used. Briefly, cDNA was synthesized using the Trancriptor One-Step RT-PCR (Roche) kit with a combination of specific primers (forward primer labeled 5Bo8254 and reverse primer 5Bo8707) yielding a final product of 454 base pair (bp) (nucleotide positions 8254-8707, H77 reference genome- GenBank accession number AF009606). Afterwards, a hemi-nested PCR (FastStart High Fidelity PCR System, dNTPack, Roche) including a fragment of 388 bp in NS5B was carried out including nucleotide positions from 8254 to 8641. Inner primers for hemi-nested PCR were forward primer 13n5Bo8254 and reverse primer 13n5Bo8641. This product was subjected to a nested PCR with multiplex identifiers (MIDs) barcoded primers, so that each patient was labeled using Roche’s validated MID. The PCR reaction was performed in 96-well microplates. All measures to prevent PCR contamination were strictly applied. PCR products were cleaned up (Ampure Agencourt RNAClean XP, Beckman Coulter) and quantified by spectrophotometry (NanoDrop, ThermoScientific).

The library preparation started by diluting amplicons to create a multiplexed library with equimolar DNA concentrations followed by PCR consisting of end repair and A-tailing. Then, 5′ and 3′adapter ligation was PCR amplified and all postreaction cleanups (Kapa Pure Beads) were performed according to the Roche KAPA Hyper Prep Protocol for Illumina libraries. Products were quantified using Quant-it PicoGreen dsDNA Assay Kit (ThermoScientific) in a Light Cycler 480 II real-time PCR machine (Roche). Then the library was normalized to a concentration of 4 nM and pooled with a concentration of 20pM and PhiX as internal control. Indexed libraries were sequenced in the Illumina MiSeq platform using the v3 chemistry.

The fasta files obtained were demultiplexed to obtain a fasta file for each sample and for the strand. Sequences were filtered using Usearch, accepting only those with quality score (Q) > 30. Finally, HCV GTs were assigned using geno2pheno and BLAST. In addition, consensus sequences for each GT obtained using Roche-NGS in both single- and mixed-GT cohorts, including consensus sequence of each HCV co-infection, were built.

The average time required to complete a run was approximately 5 hours with the Abbott RT-PCR compared to almost 3 days with the Roche-NGS test.

## Results

A total of 139 HCV-positive patients were analyzed for HCV genotyping by both methods, Abbott RT-PCR and Roche-NGS. The demographic, virological, and clinical features of the 139 patients are shown in Table 1.

**Table 1:** Demographic, virological, and clinical features.

Sex, n (%)	Single-genotype cohort	Mixed-genotype cohort	*p-value*
Male	59 (52)	9 (36)	0.23
Female	55 (48)	15 (60)	0.40
**Age, year (** ^**a**^ **IQR, interquartile range)**			
Mean	58.53 ± 14.16	62.48 ± 12.97	0.18
Median	55 (19.75)	60 (22)	
Range	26–89	43–86	
**HCV Viral load**, **log10 IU/mL (IQR)**			
Mean	6.12 ± 0.78	5.73 ± 0.80	**0**.**03**
Median	6.25 (0.96)	5.96 (1.14)	
Range	2.95–7.52	3.93–6.85	
HIV co-infected, n (%)	20 (18)	3 (12)	

Illumina sequencing provided a number of reads ranging from 14228 to 315971 per sample, and the average number of reads per sample was 80920 (standard deviation of 48976).

### Single-genotype cohort

According to the Abbott RT-PCR, for the single-GT cohort -which included 114 patients- the most commonly identified GT was 1b accounting for 37% (42 out of 114) of all samples, followed by GT 4, which accounted for 25% (29 out of 114). The GTs of 96% (109 out of 114) samples were confirmed using the Roche-NGS NS5B sequence analysis.

Although the major GT 1 was confirmed in most of the samples, Abbott RT-PCR failed to report subtypes of HCV in four samples that could be identified using Roche-NGS (three were identified as GT 1a and one as GT 1b). The samples with GT 1 and subtype not determined using Abbott RT-PCR, which were successfully subtyped using the Roche-NGS test, were scored as minor discordance in the final analysis.

All samples originally classified as GT 2 (7 out of 114) and GT 3 (8 out of 114) were confirmed using Roche-NGS.

Abbott RT-PCR and Roche-NGS were consistent in 96.15% (25 out of 26) of the GT 4 samples. One case of Abbott RT-PCR GT 4 was genotyped as GT 1a using Roche-NGS; Roche-NGS could not report results in three additional GT 4 patients (no amplification). Genotype 5 was confirmed using Roche-NGS in 3 samples; and one GT 5 sample could not be amplified using Roche-NGS. Overall, Roche-NGS could not identify the GT in four patients, since sequence information was not available for them and there was insufficient sample volume to repeat testing.

All 114 samples originally determined as single GTs using Abbot RT-PCR were confirmed to be single-GT infections using Roche-NGS. None of these samples were found to have mixed-GT infections.

Furthermore, Roche-NGS allowed better characterization of GTs identifying the subtype for all the samples that could be amplified (110 out of 114, 96%).

### Mixed-GT cohort

The mixed-GT cohort consisted of 25 selected patients that were originally identified as having double/triple HCV infections using Abbott RT-PCR.

Most mixed HCV infections (11 out of 25, 44%) were of GTs 1b + 4. Concordance between the Abbott RT-PCR and the Roche-NGS tests was found for 19 out of 25 samples for this cohort (76%). Discordances were as follow: for three patients with a mixed infection found with the Abbott assay [two (1b + 4); one (1a + 3)], the Roche-NGS test detected only the subtype 1b (100% of 120168, 127520 and 128448 reads). Very divergent results between Abbott RT-PCR and Roche-NGS were found for the other three discordant samples (1b + 4, 4 + 5, 4 + 5 + 1 subtype not determined were identified using Roche-NGS as 1a + 4d, 1a + 4f, 1a + 4f, respectively). These results are shown in Table 2.

**Table 2:** Comparison of HCV genotyping results between Roche-NGS and Abbot RT-PCR methods in 25 selected mixed-GT infected patients.

No.	Patient code	Genotyping result		
		Abbot RT-PCR	Roche-NGS (relative proportion)	Roche-NGS (reads)
1	*m2*.*a*	1a + 1b	1a (53%) + 1b (47%)	89008
2	*M7*	1a + 1b	1a (69%) + 1b (31%)	105439
3	*m23*.*b*	1b + 3	1b (65%) + 3a (35%)	88260
4	*m11*.*a*	1a + 4	1a (83%) + 4d (17%)	77923
5	*m22*.*d*	1a + 4	1a (61%) + 4a (39%)	130551
6	*m3*.*a*	1b + 4	1b (64%) + 4d (36%)	73183
7	*m4*.*a*	1b + 4	1b (40%) + 4d (60%)	57058
8	*M8*	1b + 4	1b (25%) + 4d (75%)	74392
9	*m9*.*a*	1b + 4	1b (60%) + 4d (40%)	124810
10	*m10*.*a*	1b + 4	1b (38%) + 4d (62%)	116940
11	*m13*	1b + 4	1b (84%) + 4d (16%)	91539
12	*m17*	1b + 4	1b (74%) + 4a (26%)	95147
13	*m21*	1b + 4	1b (68%) + 4d (32%)	66769
14^a^	*m5*.*a*	1 ns + 4	1a (81%) + 4d (19%)	126197
15^a^	*m12*.*a*	1 ns + 4	1a (58%) + 4 f (42%)	128524
16	*m6*.*a*	2 + 4	2c (64%) + 4d (36%)	34373
17	*m24*.*a*	2 + 4	2a (54%) + 4d (46%)	52213
18	*m15*.*b*	4 + 5	4a (27%) + 5a (73%)	59460
19	*m25*.*a*	4 + 5	4d (89%) + 5a (11%)	91186
20^b^	*M1*	1b + 4	1b (100%)	120168
21^b^	*m18*.*a*	1a + 3	1b (100%)	127520
22^b^	*m19*	1b + 4	1b (100%)	128448
23^b^	*m16*	1b + 4	1a (86%) + 4d (14%)	61381
24^b^	*m20*.*a*	4 + 5	1a (23%) + 4 f (77%)	74304
25^b^	*m14*.*b*	4 + 5 + 1 ns	1a (29%) + 4 f (71%)	29316

^a^Classified as genotype 1 subtype not determined (ns) using Abbott RT-PCR, correctly identified using Roche-NGS.

^b^Discordance of mixed HCV infection results.

After running Roche-NGS, overall concordance with the Abbot RT-PCR was 87%. Concordance was very high for the single-infection cohort (94%-minor- to 96%-overall-), and lower for the mixed-GT infection cohort (minor-68%- to 76%-overall-). The concordance rate between both assays is shown in Table 3.

**Table 3:** Concordance rates between Roche-NGS and Abbott RT-PCR.

Genotype identified using Abbott RT-PCR	Number of samples	Roche-NGS results	Concordance (Abbot RT-PCR *vs*. Roche-NGS)	
			Minor^a^	Overall
Single-genotype	114	114 single-infection	107, 94% Kappa = 0.90	109, 96% Kappa = 0.94
Mixed-genotype	25	22 mixed-infection 3 single-infection	17, 68% Kappa = 0.40	19, 76% Kappa = 0.55

^a^Abbott RT-PCR genotype 1 subtype not determined samples, which were successfully subtyped using Roche-NGS, were scored as minor discordance.

**Figure 1:**
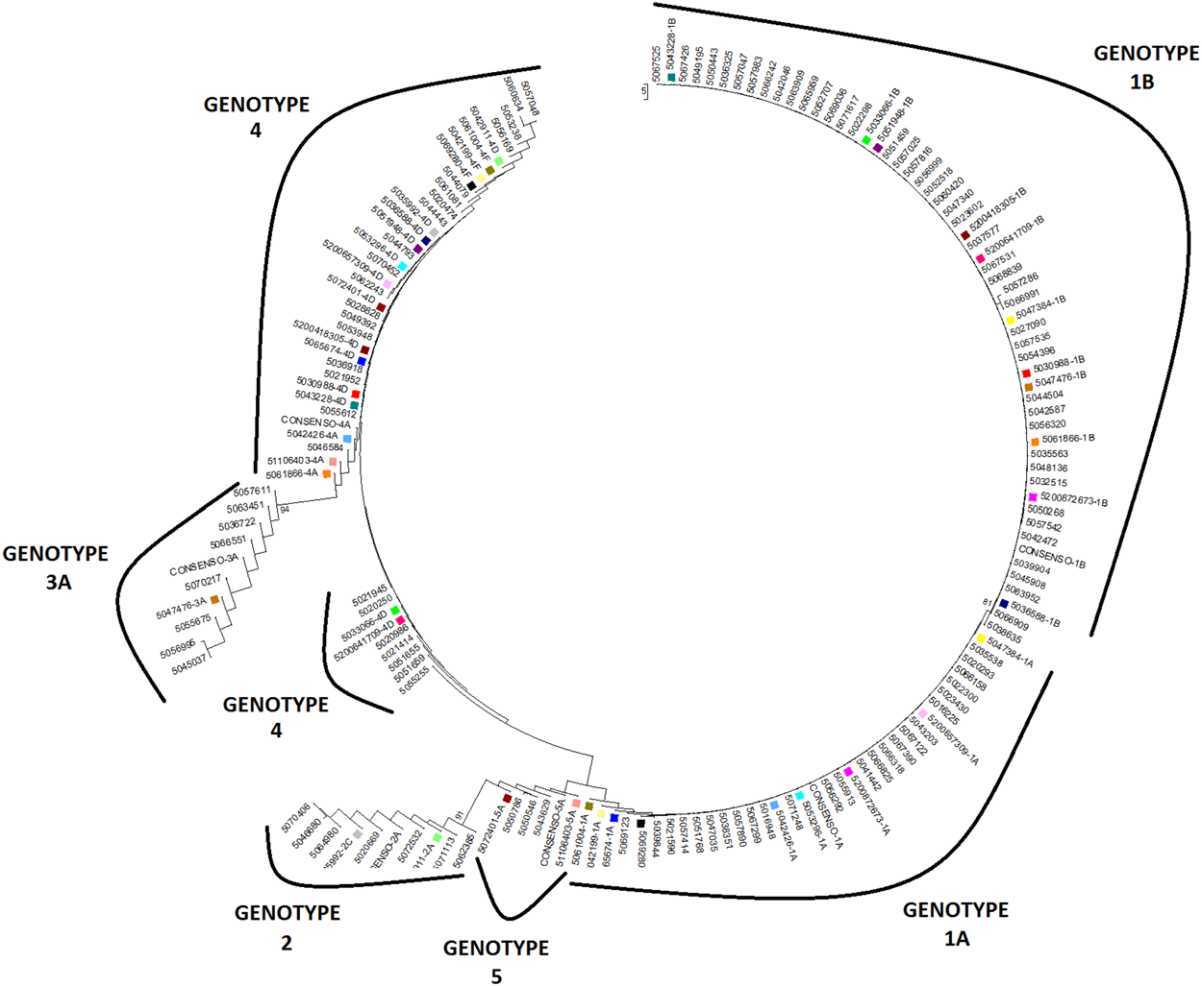
Phylogenetic tree of consensus sequences built using data generated by the Roche-NGS for all patients in both single- and mixed-GT cohorts. Each coloured square represents a mixed-GT infection.

In addition, we performed a thorough phylogenetic analysis of all the consensus sequences found in all the studied GTs to rule out the possibility of cross-contamination between samples in our Roche-NGS experiments. As shown in Fig. 1, no cross-contamination was found.

## Discussion

At present, HCV genotyping is still used in clinical practice and remains vital to determine the most appropriate antiviral regimen and treatment duration for each patient as well as for epidemiological studies^6,24^. In fact, HCV genotyping is recommended for the initial evaluation of HCV infected patients by international clinical guidelines^25,26^. The impact of mixed-GT infections on clinical care is not fully understood, but its presence may play an important role for treatment initiation when pangenotypic regimens are not available or are not going to be used. Although HCV infection by mixed GTs has been frequently reported^3,10,27–30^, the true incidence of HCV mixed infections is not yet clear because commercial genotyping assays are not intended to shed light on this issue and because the detection rate of mixed infections is highly variable depending on the methodology used and the geographic area. In our study, we describe how the Abbott RT-PCR genotyping assay performs compared to a Roche-NGS prototype. We have found a very good concordance between both assays for patients that are monoinfected with a single genotype. We have found a moderate concordance with Roche-NGS when Abbott RT-PCR reported a mixed infection.

The Abbott RT-PCR assay is an easy handling, high-throughput, automated HCV genotyping method that can identify the GT in a short time. Admittedly, the main limitation of the Abbott RT-PCR assay and other currently available routine HCV genotyping assays is the inability to identify subtypes other than 1a and 1b. In addition, Abbott RT-PCR fails to subtype GT 1 samples occasionally. According to our results, most of those GTs 1 subtype not determined were identified as GT 1a when using Roche-NGS. Roche-NGS is considered a useful tool to detect minor viral variants because it generates millions of reads in a single run^31^, so it may help to rule out the incorrect assignment of mixed infections by routine methods. However, Roche-NGS protocols are very laborious and time consuming, and are difficult to implement, so far, in the routine laboratory. In addition, Roche-NGS targets only the NS5B region^32,33^, therefore, HCV infections that are due to HCV recombinants may be missed.

Overall, we have observed a high concordance between Roche-NGS and Abbott RT-PCR (Kappa = 0.88). Only seven cases were miss-genotyped in our whole study, comprising both cohorts. Nevertheless, when we analyzed the data for the mixed-GT cohort, concordance between both assays was not so good. Discordances concentrate in the mixed-GT cohort. In fact, the kappa value in this cohort falls to 0.40 and the overall concordance ranges from 68% to 76%. In spite of these results, we propose that, when possible, mixed-GT infections identified using Abbot RT-PCR should be reconfirmed with a second assay based on Roche-NGS. Although not proven in our study, it is plausible that this recommendation could be also applied for other commercial genotyping assays. Since Roche-NGS of a fragment of the NS5B region has been reported to be the ideal method, we have not considered doing a third genotyping technique^23,31,34^.

Our study has several limitations: first, this is a retrospective study, so selection bias cannot be avoided. However, as the prevalence of mixed-GT infections is generally low in the Spanish population^23^ we had to choose this design. Second, samples were stored at −80 °C until Roche-NGS testing, so we cannot rule out that some of the discordances may be due to a loss of RNA viability during storage.

Although NGS is becoming increasingly accessible and affordable, this methodology is still not the main tool for diagnosis at routine laboratories. Given the fact that mixed-GT infections are not so prevalent, and that the Abbott RT-PCR has performed excellently at the single infected cohort, we believe that this is a suitable and reliable tool for genotyping HCV, and for the diagnosis of possible mixed HCV GT infections. However, in spite of our results for the mixed-GT infection cohort, when possible, we recommend the use of a NGS assay to confirm mixed-GT infection results obtained using Abbott RT-PCR to achieve an optimal management of HCV patients.
